# Seasonal Variation Drives Leaf Anatomical Structure, NSC Allocation, and C:N:P Stoichiometric Characteristics in *Ochroma lagopus* Plantations

**DOI:** 10.3390/plants15152350

**Published:** 2026-07-30

**Authors:** Yueqiao Han, Yuanxi Liu, Guihe Duan, Guanben Du, Rui Shi, Junwen Wu

**Affiliations:** 1 College of Forestry, Southwest Forestry University, Kunming 650224, China; 2 Dali Branch, Yunnan Institute of Forestry and Grassland Survey and Planning, Dali 671000, China; 3 College of Materials and Chemical Engineering, Southwest Forestry University, Kunming 650224, China; 4 Yunnan Provincial Key Laboratory for Conservation and Utilization of In-Forest Resource, Southwest Forestry University, Kunming 650224, China

**Keywords:** *Ochroma lagopus*, introduced cultivation, dry wet season, leaf anatomical structure, non-structural carbohydrates, C:N:P stoichiometry, tropical plantation

## Abstract

*Ochroma lagopus* is a fast-growing, low-density broadleaf tree species with increasing importance for tropical plantation forestry and lightweight bio-based materials. However, juvenile plantations may be sensitive to seasonal changes in water availability and soil nutrient status, particularly in tropical monsoon regions with strong drywet season transitions. In this study, two-year-old *O. lagopus* plantations in Xishuangbanna, Yunnan Province, China, were used to compare leaf anatomical structure, non-structural carbohydrate (NSC) components in leaves and roots, and C:N:P stoichiometric characteristics between the wet and dry seasons. Correlations among leaf structure, carbon allocation, and nutrient stoichiometry were also analyzed. The results showed significant seasonal differences in leaf anatomical structure. Leaves in the wet season had more developed vascular bundles and midrib tissues, whereas leaves in the dry season had deeper substomatal chambers and higher tissue looseness. NSC components were mainly affected by organ differentiation, whereas seasonal effects were weak. Roots maintained relatively high and stable carbohydrate levels. C:N:P stoichiometric characteristics were affected by both season and organ, and root P concentration and P-related ratios were more sensitive to seasonal transition. Leaf and root N/P ratios were generally below 14, indicating a nutritional status associated with relative N limitation. Correlation analysis showed that leaf anatomical traits were significantly correlated with leaf NSC, C, N, and P concentrations and stoichiometric ratios, revealing a coordinated structural carbon nutrient response of *O. lagopus* during the dry wet season transition. These findings suggest that the response of juvenile *O. lagopus* plantations to seasonal environmental variation is not driven by water availability alone, but is jointly regulated by leaf structural plasticity, root carbon pool stability, nutrient leaching during the wet season, and P availability in red soil. This study provides a theoretical basis for the introduction and cultivation, nutritional diagnosis, and seasonal management of juvenile *O. lagopus* plantations in tropical regions.

## 1. Introduction

*Ochroma lagopus* is native to tropical America and is one of the fastest-growing commercial broadleaf tree species with the lowest wood density worldwide. Its wood combines low density, high specific strength, good thermal insulation, and favorable processing properties, and it has long been used in aerospace, shipbuilding, and lightweight structural materials. In recent years, the rapid increase in demand for wind turbine blade core materials and renewable bio-based materials has increased the strategic value of *O. lagopus* [1,2,3]. Because natural resources are limited, plantation cultivation has become an important pathway to ensure a continuous supply of raw material. Tropical and subtropical regions in China provide a suitable climatic basis for the introduction and cultivation of *O. lagopus*. However, as an introduced fast-growing tree species, its seasonal adaptability, growth stability, and nutrient demand in introduced regions remain poorly understood. Juvenile trees have high growth rates and strong carbon and nutrient demands. Their sensitivity to fluctuations in water and soil nutrient availability may directly affect subsequent stand productivity. Therefore, evaluating the responses of juvenile *O. lagopus* plantations to seasonal environmental variation based on structural and ecophysiological traits is essential for assessing introduction adaptability and improving cultivation practices.

Xishuangbanna has a typical tropical monsoon climate, with concentrated rainfall in the wet season and a marked decrease in precipitation during the dry season. Water supply fluctuates strongly within the year. The wet season has traditionally been regarded as a period of sufficient water availability and favorable growth, whereas the dry season is often considered a period of water stress [4]. Recent studies, however, have shown that the effects of tropical seasonal environments on trees are not driven by water availability alone. Instead, they reflect the combined effects of water supply, light conditions, vapor pressure deficit, nutrient transport, and rhizosphere processes [5,6]. Rainfall during the wet season improves soil moisture and promotes nutrient diffusion, but it also enhances leaching, leading to losses of nitrate N, dissolved organic N, and base cations. Leaching is reduced in the dry season, but soil drying limits nutrient diffusion and root uptake efficiency [5]. In acidic red soil, P is readily immobilized by Fe/Al oxides, and its availability is jointly regulated by soil moisture, pH, and rhizosphere processes [7]. Thus, the seasonal response of *O. lagopus* in this region should not be simplified as “growth promotion in the wet season and stress in the dry season”. Instead, it should be understood within a framework that integrates seasonal water fluctuation, nutrient leaching during the wet season, P fixation in red soil, and plant functional differentiation.

Leaves are the primary interface for material and energy exchange between plants and the external environment. Their anatomical structure is highly sensitive to water availability, light environment, and nutrient conditions [8,9]. Traits such as leaf thickness, cuticle, epidermal cells, palisade tissue, spongy tissue, substomatal chamber, and vascular bundles jointly determine light interception, gas diffusion, water retention, and material transport capacity [10]. Previous studies often regarded a thicker cuticle, developed epidermis, and compact mesophyll as structural bases for drought or high-light tolerance. Recent plant functional trait research, however, has emphasized the multifunctional nature of leaf anatomical traits [11,12]. Vascular bundles are not only involved in water transport but also affect mineral nutrient supply and photosynthate export. Palisade tissue and spongy tissue influence photosynthetic area and CO_2_ diffusion, and are closely related to leaf N and P allocation and carbon assimilation capacity [13]. Therefore, leaf anatomical structure should not be treated only as a morphological descriptor. It should be considered a structural trait linking environmental resource supply, leaf functional development, and carbon-nutrient metabolism. For fast-growing introduced species such as *O. lagopus*, rapid juvenile growth may impose high demands on the development of leaf conducting structures and mesophyll tissues. Seasonal plasticity in leaf anatomical structure during the dry wet season transition may therefore directly reflect its seasonal adaptability.

Non-structural carbohydrates (NSCs) are key indicators of plant carbon economy and mainly include soluble sugars and starch [14,15]. In recent years, NSC research has shifted from simply describing carbon storage to explaining source sink relationships, inter-organ carbon allocation, stress buffering, and post-stress recovery [16]. Soluble sugar is an active carbon form involved in transport and metabolism, and also participates in osmotic adjustment and stress responses. Starch represents a more stable carbon reserve. Plant NSC levels are jointly affected by photosynthetic input, growth consumption, respiratory demand, organ storage, and assimilate transport [16]. In fast-growing tree species, improved water availability in the wet season may enhance carbon assimilation, but rapid growth also increases carbon consumption and export. Thus, NSC does not necessarily accumulate significantly during the wet season. In contrast, belowground organs such as roots may maintain relatively stable NSC pools to support basal metabolism in the dry season, fine-root renewal, and seasonal growth recovery [17]. Therefore, analysis of NSC components in *O. lagopus* needs to consider both seasonal effects and organ differentiation, rather than inferring carbon economic status only from mean differences between the wet and dry seasons.

Plant C:N:P stoichiometric characteristics provide an important framework for understanding the relationship between carbon accumulation and nutrient allocation. C mainly reflects organic carbon accumulation and structural material formation. N is closely associated with proteins, enzymes, chlorophyll, and photosynthetic capacity. P participates in ATP, nucleic acids, membrane structure, and energy metabolism [9,18,19]. The C/N, C/P, and N/P ratios are often used to evaluate nutrient use characteristics and potential nutrient limitation. However, recent ecological stoichiometry studies have also emphasized that N/P thresholds should not be applied mechanically to diagnose nutrient limitation [20,21]. This is especially important in highly weathered tropical soils, strongly leached environments, and introduced fast-growing plantations, where plant tissue N/P is jointly affected by soil nutrient availability, root uptake, organ allocation, growth dilution, and seasonal water processes [9,22]. In the red soil region of Xishuangbanna, N leaching during the wet season and P fixation in acidic soil may occur simultaneously, and their effects on plant nutritional status are not necessarily synchronized. Combining C:N:P stoichiometric characteristics with NSC components can therefore help determine whether *O. lagopus* responds to the dry wet season transition mainly through changes in carbon reserves or through nutrient ratio reorganization.

Current studies on *O. lagopus* have mainly focused on seedling propagation, cultivation techniques, growth performance, and wood properties [23,24,25]. However, the coordinated responses of leaf anatomical traits, carbon allocation and nutrient stoichiometry to seasonal environmental variation remain poorly understood, especially in juvenile plantations established in tropical monsoon regions. Consequently, an important knowledge gap remains regarding how leaf structural traits, organ-specific carbon reserves, and nutrient stoichiometry covary under contrasting wet and dry conditions in juvenile *O. lagopus* plantations. Addressing this gap requires an integrated whole-plant trait approach rather than analysis of individual indicators.

In this study, we investigated two-year-old *O. lagopus* plantations in Xishuangbanna, southwest China. Leaf anatomical traits, NSC components and C:N:P stoichiometric characteristics of leaves and roots were compared between the wet and dry seasons. Correlation analysis and PCA were used to evaluate the coordination among structural, carbon, and nutrient traits. We addressed three questions: (1) which leaf anatomical traits show seasonal plasticity during the dry wet season transition; (2) whether NSC components and C:N:P stoichiometric characteristics show similar seasonal and organ-level responses; and (3) whether leaf anatomical structure, NSC allocation and C:N:P stoichiometry form a coordinated trait response. The novelty of this study lies in integrating leaf anatomical plasticity with above- and belowground carbon reserves and nutrient stoichiometry within a juvenile tropical plantation. This approach extends previous *O. lagopus* research beyond single-trait and cultivation-focused assessments and may provide a useful framework for other fast-growing tropical plantation species. 

## 2. Materials and Methods

### 2.1. Study Area

The study site was located in an *O. lagopus* plantation at Mengxing Farm, Mengla County, Xishuangbanna, Yunnan Province, China (21°54′–21°55′ N, 101°10′–101°30′ E). The elevation ranges from 540 to 650 m. The region has a subtropical rainforest monsoon climate, with a mean annual temperature of 21.5 °C and mean annual precipitation of 1600 mm. The soil is dominated by red soil, with a pH of 4.28, organic matter of 23.6 g kg^−1^, total N of 1.59 g kg^−1^, total P of 0.32 g kg^−1^, and total K of 12.0 g kg^−1^. Mengla County is located in a tropical monsoon rainforest climate zone with distinct dry and wet seasons. The dry season lasts from November to April of the following year. During the dry season, local precipitation is low and sunshine duration is relatively long, and plant growth is susceptible to seasonal drought. Two-year-old *O. lagopus* plantations were used in this study.

### 2.2. Experimental Design and Sample Collection

Three 10 m × 10 m plots with similar site conditions and stand structure were established in the *O. lagopus* plantation. Each plot was used as an independent spatial sampling unit. In each plot, three two-year-old trees with similar growth status and no obvious disease or pest damage were selected, resulting in a total of nine labeled sample trees. Trees were spatially nested within plots, and the same nine trees were sampled during both sampling campaigns to ensure one-to-one correspondence between wet- and dry-season observations. Mean tree height was 8.24 ± 0.35 m and mean diameter at breast height was 10.67 ± 0.71 cm. Samples were collected in August 2022, representing the typical wet season, and in April 2023, representing the typical dry season.

Leaf samples were collected using a pole pruner. Mature, intact, and healthy leaves were sampled from the upper-middle canopy at approximately two-thirds of tree height. For leaf anatomical observation, two to three leaf segments of 0.5 cm × 0.5 cm were cut from both sides of the midrib at approximately one-third of the distance from the leaf apex. The segments were rinsed with PBS buffer, immediately placed in 2.5% glutaraldehyde fixative, stored in the dark, and fixed at 4 °C. Leaf samples used for NSC and C:N:P measurements were cleaned to remove surface impurities. Root samples were collected by excavation. Fresh fine roots were sampled from the 0–10 cm soil layer around the sampled trees, cleaned of attached soil and impurities, and transported to the laboratory. Leaf and root samples were heated at 120 °C for 30 min to deactivate enzymes, dried at 80 °C to constant mass, ground, sieved, and stored in sealed bags for determination of soluble sugar, starch, total NSC, and C, N, and P concentrations.

### 2.3. Measurements

#### 2.3.1. Leaf Anatomical Structure

Fixed leaf samples were dehydrated, cleared, infiltrated with paraffin, and embedded. Paraffin sections were cut at 8–10 μm thickness. Sections were stained with safranin-fast green, mounted with neutral balsam, and observed and photographed under a light microscope (Eclipse Ci-L, Tokyo, Japan). Leaf anatomical traits were measured using ImageJ v1.53r or another image analysis software. Measurements in the lamina were taken from flat regions, avoiding the midrib and large lateral veins. The measured traits included leaf thickness (LT), upper cuticle thickness (UCT), lower cuticle thickness (LCT), upper epidermis thickness (UET), lower epidermis thickness (LET), palisade tissue thickness (PMT), spongy tissue thickness (SMT), and substomatal chamber depth (SCD). Xylem area of vascular bundles (XA) and midrib vascular bundle area (MVBA) were measured in the midrib region. For each sample, intact and clearly stained sections were selected, and multiple fields of view or measurement points were randomly chosen from each section. The mean value was used as the anatomical value for that sample. The derived traits were calculated as follows:

Leaf tissue compactness (LTC) = palisade tissue thickness/leaf thickness × 100%

Leaf tissue looseness (LTL) = spongy tissue thickness/leaf thickness × 100%

Palisade/spongy ratio (P/S) = palisade tissue thickness/spongy tissue thickness

#### 2.3.2. Determination of Soluble Sugar, Starch, C, N, and P Concentrations

In this study, NSC refers to the sum of soluble sugars and starch. Samples (0.05 g) were ground and mixed with 10 mL of distilled water; the mixture was then centrifuged at 4000 r·min^−1^ for 10 min after 10 min in a boiling water bath. The supernatant was then subjected to the anthrone method using a UV-Vis spectrophotometer (HD-UV90; Shandong Holder Electronic Technology Co., Ltd., Tai’an, China). The absorbance value of soluble sugars at 625 nm was determined, and their content was calculated; the starch content in the samples was determined using the precipitate. Total C concentration was determined using the potassium dichromate sulfuric acid oxidation method. Total N concentration was determined using Nessler’s colorimetric method. Total P concentration was determined using the molybdenum antimony colorimetric method [9,17,22].

### 2.4. Data Processing and Statistical Analysis

Raw data were organized using Excel 2021, and statistical analyses were performed using SPSS 26.0 and R 4.3.0. Figures were generated using Origin 2021, GraphPad Prism 8.0, and R. Nine two-year-old trees were sampled in this study. The same trees were sampled in both the wet and dry seasons, and each tree was individually labeled to ensure one-to-one correspondence between seasonal observations. Therefore, seasonal differences were analyzed using paired statistical tests. Results are presented as means ± standard errors, and exact sample sizes are reported for each analysis. Statistical significance was set at *p* < 0.05, and high significance was set at *p* < 0.01. Finally, we performed correlation analysis and principal component analysis on the various indicators.

## 3. Results

### 3.1. Comparison of Leaf Anatomical Traits Between the Wet and Dry Seasons

**Figure 1:**
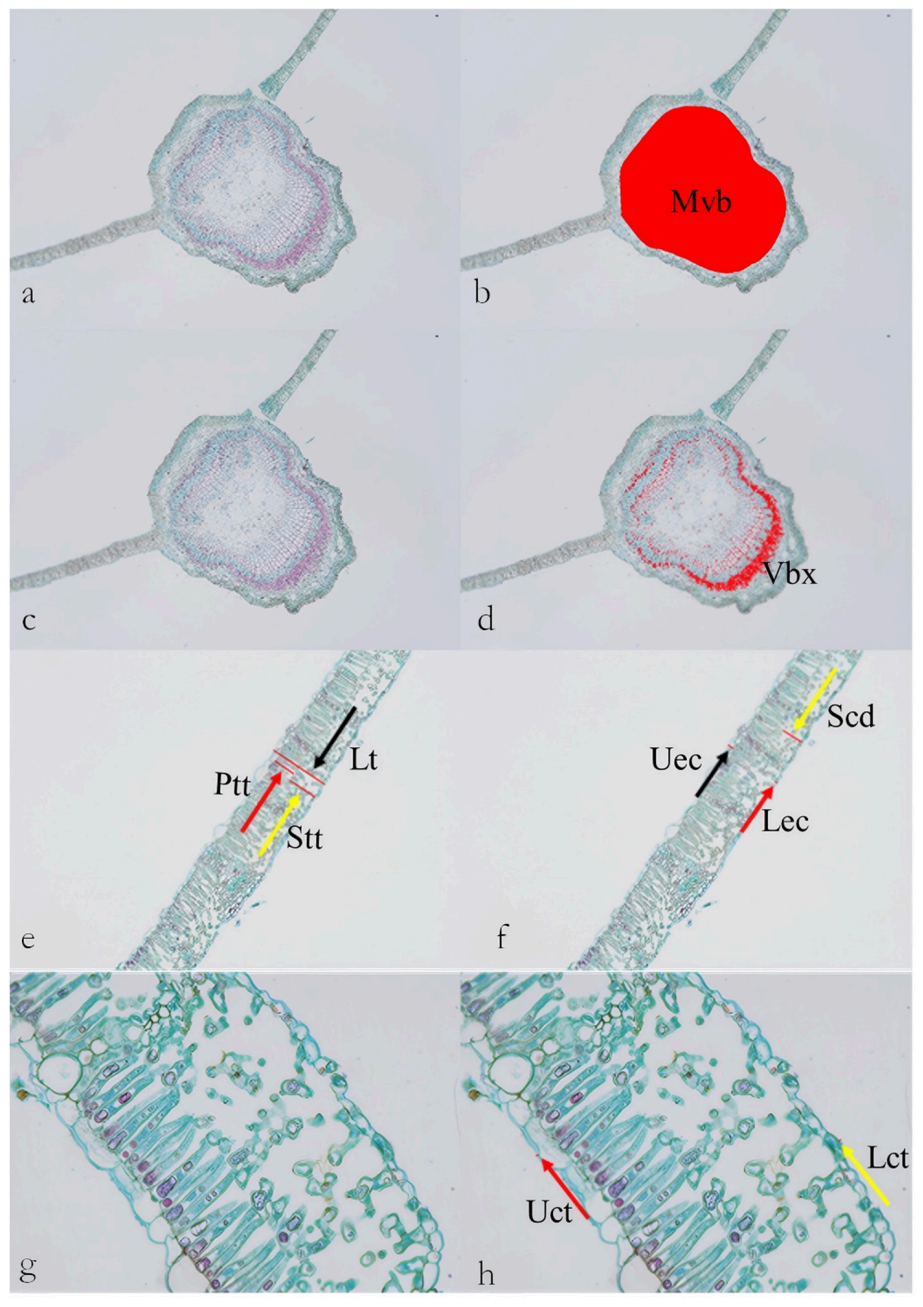
Leaf cross sections of Ochroma lagopus. Note: Panels (**a**,**c**,**e**,**g**) show dry-season samples, and panels (**b**,**d**,**f**,**h**) show wet-season samples. LT, leaf thickness; UCT, upper cuticle thickness; LCT, lower cuticle thickness; UET, upper epidermis thickness; LET, lower epidermis thickness; SCD, substomatal chamber depth; PMT, palisade tissue thickness; SMT, spongy tissue thickness; XA, xylem area of vascular bundles; MVBA, midrib vascular bundle area.

The leaf anatomical structure of *O. lagopus* consisted of the epidermis, vascular bundles, and ground tissue (Figure 1). Stellate hairs were present on the abaxial leaf surface. Epidermal cells were densely arranged, the cuticle was thin, and palisade tissue was well developed. As shown in Table 1, UCT, SCD, XA, and MVBA differed highly significantly between the wet and dry seasons (*p <* 0.01), and LTL differed significantly (*p <* 0.05). UCT, LCT, LET, PMT, P/S, LTC, XA, and MVBA were higher in the wet season than in the dry season.

### 3.2. Comparison of Plasticity in Leaf Anatomical Structure Between the Wet and Dry Seasons

As shown in Table 2, except for UCT, UET, and XA, the coefficients of variation and plasticity indices of leaf anatomical traits were higher in the dry season. In both seasons, XA had the highest coefficient of variation and plasticity index. In the wet season, PMT and LTL had the lowest coefficients of variation (0.06), and PMT had the lowest plasticity index (0.16), followed by LTL (0.19). In the dry season, LTL had the lowest coefficient of variation (0.09), and LTC and LTL had the lowest plasticity indices (0.26).

### 3.3. Effects of Organs on NSC Components and C:N:P Stoichiometric Characteristics Revealed by Two-Way ANOVA

Two-way ANOVA showed that NSC components in *O. lagopus* were mainly affected by organ (Table 3). The main effects of organ on soluble sugar, total NSC, and SS/ST ratio were highly significant (*p <* 0.001), whereas the main effects of season and the season × organ interaction were not significant (*p* > 0.05). Starch concentration was not significantly affected by season, organ, or their interaction (*p* > 0.05). C:N:P stoichiometric characteristics were affected by both season and organ. Season had significant or highly significant effects on C and P concentrations and on C/N, C/P, and N/P ratios, but had no significant effect on N concentration. Organ had highly significant effects on all C:N:P parameters (*p <* 0.001). The season × organ interaction was highly significant only for the C/P ratio (*p <* 0.001).

### 3.4. NSC Concentrations in Different Organs of O. lagopus

**Figure 2:**
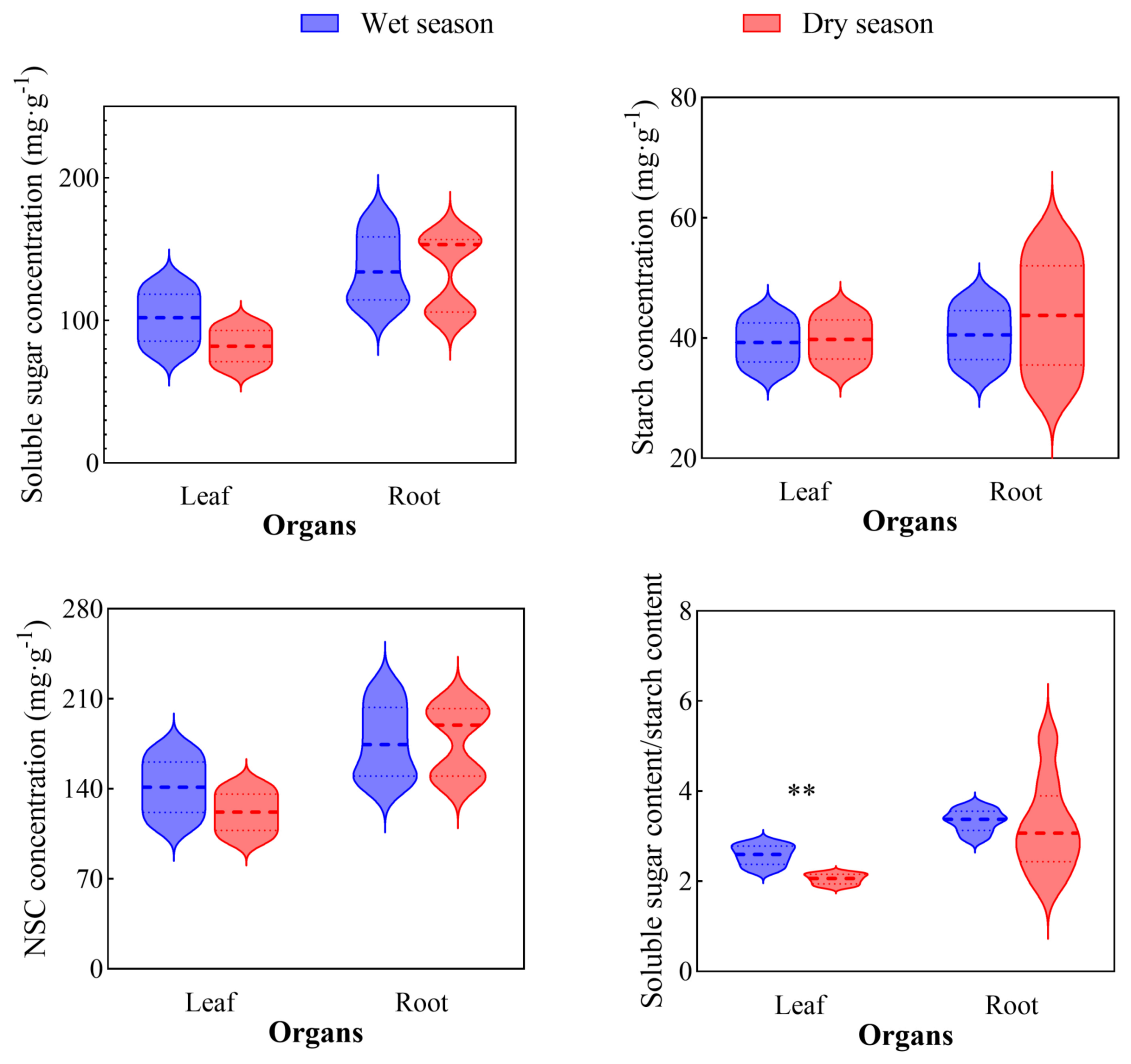
Seasonal variation in NSC components of *O. lagopus*. Asterisks indicate significant differences between the wet and dry seasons within the same organ (** *p <* 0.001).

Overall, NSC components showed stronger organ differences than seasonal differences. Root soluble sugar, total NSC, and SS/ST ratio were generally higher than those in leaves. Some leaf NSC parameters tended to increase in the wet season, but seasonal differences were not significant (Figure 2). Root soluble sugar concentration, total NSC, and SS/ST ratio were significantly higher than those in leaves (*p <* 0.05), whereas starch concentration did not differ significantly between leaves and roots (*p* > 0.05). These patterns indicate that variation in NSC components was mainly associated with organ differences rather than seasonal changes. Organ-specific comparisons showed that leaf NSC-related parameters did not differ significantly between the wet and dry seasons. Leaf SS/ST ratio was higher in the wet season than in the dry season, with an increase of 25.85%. Leaf soluble sugar and total NSC concentrations increased by 24.39% and 15.99%, respectively, in the wet season compared with the dry season, but these differences were not significant (*p* > 0.05). Root NSC-related parameters did not differ significantly between the two seasons (*p* > 0.05), and seasonal fluctuations were small.

### 3.5. C:N:P Stoichiometric Characteristics in Different Organs of O. lagopus

**Figure 3:**
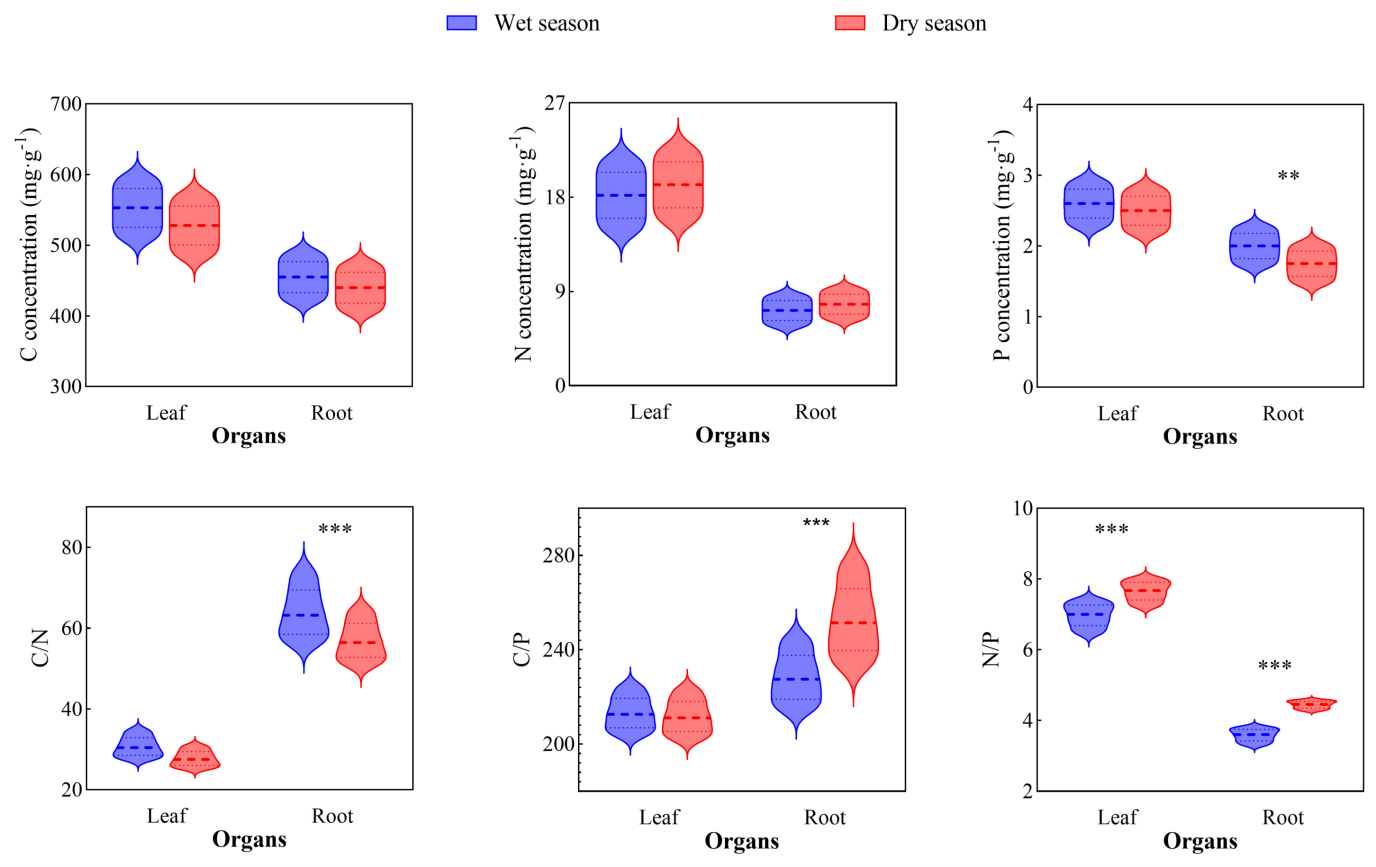
Changes in C, N, and P contents in different organs of Ochroma lagopus in the wet and dry seasons. (** *p* < 0.01; *** *p* < 0.001).

C:N:P stoichiometric characteristics in *O. lagopus* were affected by both organ and season. Leaves mainly showed a seasonal difference in N/P ratio, whereas roots mainly showed seasonal differences in P concentration and P-related ratios (Figure 3). Leaf C, N, and P concentrations and N/P ratio were significantly higher than those in roots (*p <* 0.05), whereas leaf C/N and C/P ratios were significantly lower than those in roots (*p <* 0.05). Organ-specific comparisons showed that leaf C:N:P stoichiometric parameters were generally stable between the wet and dry seasons, with only the N/P ratio being significantly higher in the dry season, increasing by 9.74%. Root stoichiometric parameters responded more strongly to seasonal variation than leaf parameters. Root P concentration and C/N ratio were significantly higher in the wet season than in the dry season. In contrast, root C/P and N/P ratios were significantly higher in the dry season than in the wet season. Root C and N concentrations did not differ significantly between the two seasons (*p* > 0.05).

### 3.6. Correlations Among Leaf Anatomical Structure, NSC Components, and C:N:P Stoichiometric Characteristics in the Wet and Dry Seasons

**Figure 4:**
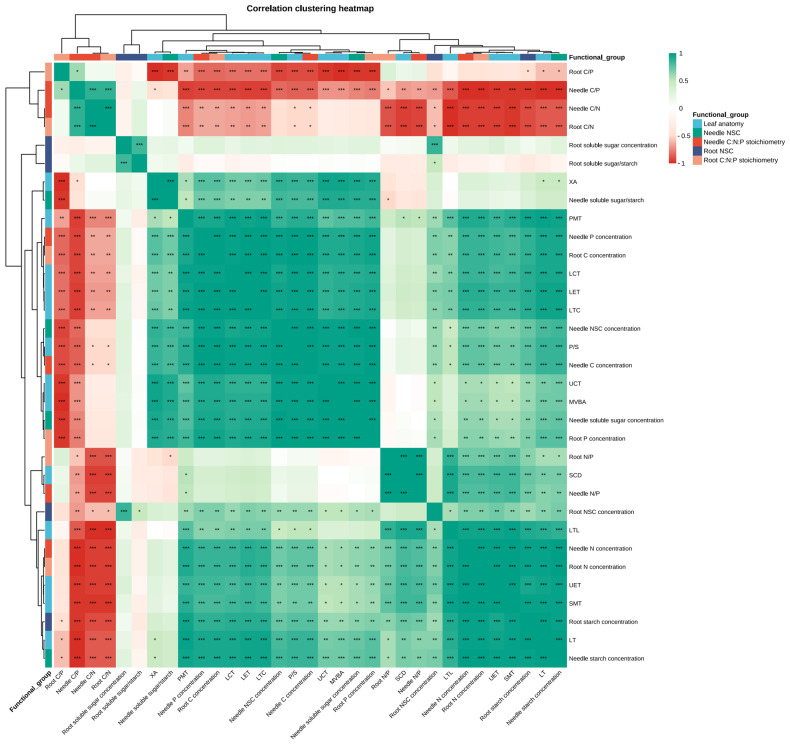
Correlation analysis of leaf anatomical structure, NSC components, and C:N:P stoichiometric characteristics of *O. lagopus* in the wet and dry seasons. (* *p* < 0.05; ** *p* < 0.01; *** *p* < 0.001).

Spearman correlation analysis showed distinct patterns of statistical association among leaf anatomical traits, NSC components, and C:N:P stoichiometric characteristics (Figure 4). Most leaf anatomical traits, including LT, PMT, SMT, and LCT, clustered together and were positively correlated, indicating coordinated variation in leaf structural characteristics. LT, PMT, and SMT were generally positively associated with leaf starch, NSC, and C, N, and P concentrations, but were negatively associated with leaf C/N and C/P ratios. P/S, XA, and MVBA were associated with leaf soluble sugar concentration, NSC concentration, and the soluble sugar-to-starch ratio, indicating covariation between vascular–mesophyll traits and carbohydrate-related variables. Leaf and root C:N:P stoichiometric traits also showed coordinated covariation. Leaf C, N, and P concentrations were positively correlated with the corresponding root concentrations, and similar patterns were observed for C/N and N/P ratios. In contrast, leaf soluble sugar concentration and the soluble sugar-to-starch ratio showed relatively weak associations with the corresponding root traits, suggesting weaker coordination of soluble carbohydrate status between leaves and roots. Root NSC and starch concentrations were primarily associated with root nutrient status and showed weaker cross-organ associations with leaf NSC components.

Overall, leaf anatomical traits, carbohydrate status, and nutrient stoichiometry showed coordinated statistical associations in *O. lagopus* seedlings. These relationships describe patterns of covariation among traits but do not establish physiological coupling or causal relationships.

### 3.7. Principal Component Analysis of Leaf Anatomical Structure, NSC Components, and C:N:P Stoichiometric Characteristics in the Wet and Dry Seasons

**Figure 5:**
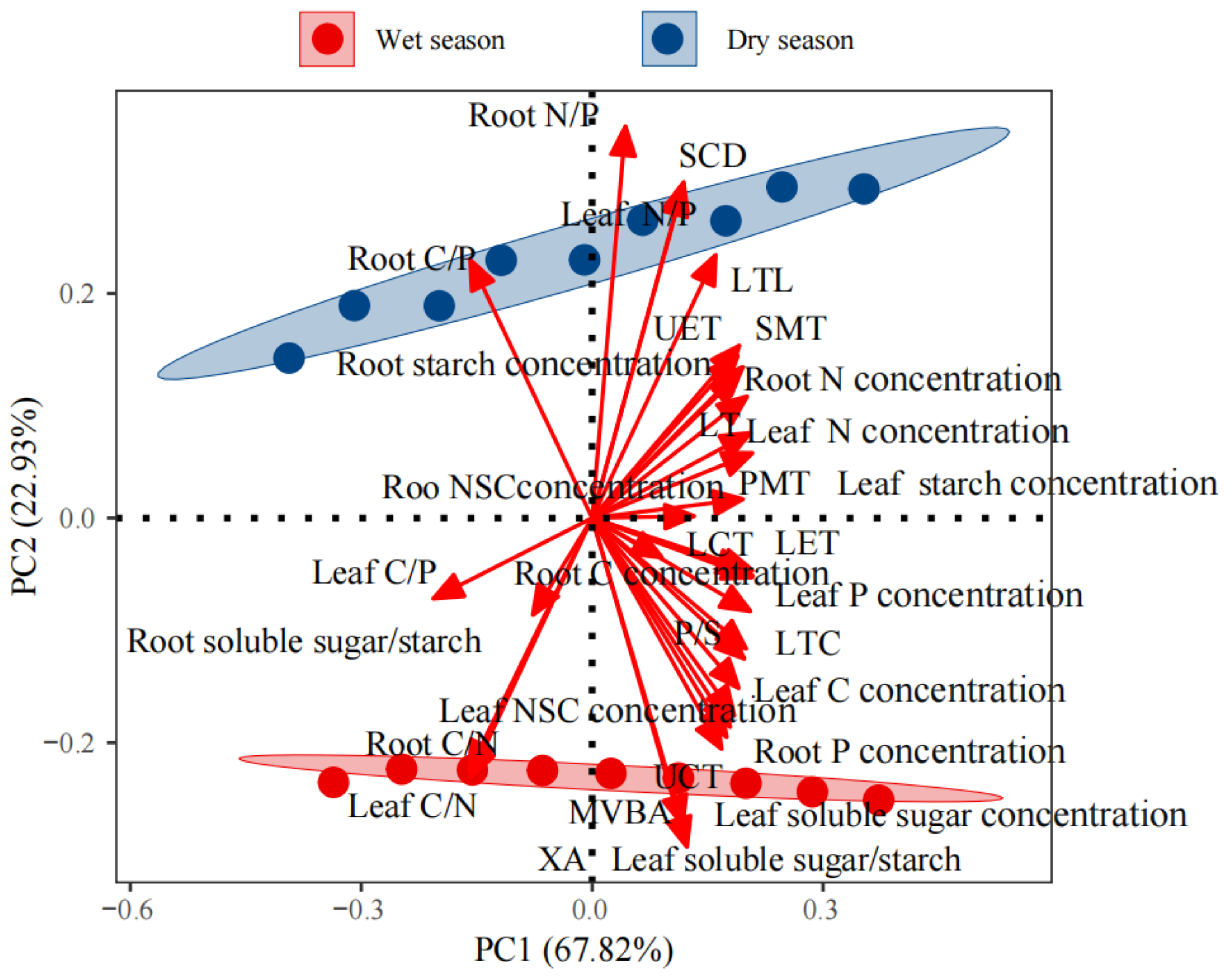
PCA of leaf anatomical structure, NSC components, and C:N:P stoichiometric characteristics of *O. lagopus* in the wet and dry seasons. Red and blue points represent wet-season and dry-season samples, respectively. Ellipses indicate confidence regions for samples from different seasons. Arrows indicate the direction and contribution of trait variables in the PCA ordination space.

Principal component analysis (PCA) showed that PC1 and PC2 explained 67.82% and 22.93% of total variance, respectively, and PC1 and PC2 together explained 90.75% of total variance (Figure 5). Wet-season and dry-season samples were clearly separated in the ordination space. Wet-season samples were mainly located on the negative side of PC2, whereas dry-season samples were mainly located on the positive side of PC2. This separation indicated clear seasonal differences in the integrated trait combinations of leaf structure, carbohydrates, and stoichiometry. Variable ordination showed that wet-season samples were closely associated with UCT, XA, MVBA, leaf soluble sugar, leaf SS/ST ratio, and root P. Dry-season samples were closely associated with SCD, LTL, SMT, leaf N/P ratio, root N/P ratio, root C/P ratio, and root starch. PC1 mainly represented a gradient in leaf structural development and elemental concentrations, whereas PC2 mainly represented seasonal differentiation in trait combinations. Overall, the separation between wet- and dry-season samples in the PCA ordination space was associated with variation in leaf anatomical traits, leaf NSC allocation, and root P-related stoichiometric parameters.

## 4. Discussion

### 4.1. Plastic Regulation of Leaf Anatomical Structure During the Dry Wet Season Transition

Leaf anatomical structure provides a structural basis for tree responses to seasonal variation in water availability, light conditions, and nutrient environment [13]. In this study, leaves in the dry season had higher SCD and LTL, most anatomical traits showed higher variability in the dry season, and XA showed high plasticity in both seasons (Table 2). For two-year-old *O. lagopus* plantations in Xishuangbanna, the wet and dry seasons differ not only in water availability but also in radiation conditions, vapor pressure deficit, soil nutrient transport, and plant growth rate [4,6]. Therefore, the observed seasonal differences in leaf structure likely represent the combined influence of water availability, leaf development, and resource transport demand, although the potential confounding of seasonal and interannual effects in our sampling design prevents a strict attribution to drought alone. These patterns, though interpreted cautiously, align with the view emerging from southern Amazonian forests that leaf and xylem anatomical variation under different water regimes involves multiple hydraulic-functional strategies, rather than conforming to a single universal drought response [26]. During the wet season, abundant precipitation increases soil moisture and improves root water uptake and mineral nutrient transport, and *O. lagopus* enters a more active growth phase. The vascular system supports not only water conduction but also mineral nutrient transport and photosynthate export. For fast-growing broadleaf species such as *O. lagopus*, rapid growth during the wet season increases leaf demand for water, nutrients, and assimilate transport. More developed vascular structures therefore likely provide structural support for conducting capacity during periods of active growth. Thus, the key implication of wet-season leaf structural changes is not simply enhanced stress resistance, but a better match among water supply, nutrient input, and carbon export capacity during rapid growth [27]. In previous studies, a thicker cuticle has often been interpreted as a structure that enhances drought tolerance or reduces water loss. In tropical monsoon regions, however, wet-season leaves may also experience strong radiation, high temperature, high humidity, and pathogen pressure [28,29]. A more developed cuticle may be related to leaf maturity, mechanical protection, reduction in surface water film effects, and protection against biotic and abiotic disturbance. Therefore, increased cuticle development in the wet season should not be interpreted only as drought adaptation. It more likely represents a multifunctional protective structure formed during rapid leaf development in *O. lagopus*.

During the dry season, reduced precipitation decreases soil water supply, which may affect leaf water status, cell turgor, and tissue development. Increases in SCD and spongy tissue air spaces may alter internal gas diffusion pathways, but may also reflect reduced compactness of mesophyll development. This loosening of leaf structure does not necessarily indicate stronger adaptation. It may also reflect structural consequences of water-limited leaf development during the dry season. From a plasticity perspective, greater inter-individual variation in leaf structure during the dry season may result from uneven water availability, surface soil drying, and reduced effective root water uptake zones, which amplify microenvironmental differences among individuals. In contrast, water limitation is alleviated during the wet season, and leaf structure may more strongly converge toward functional development required for rapid growth. The high plasticity of XA in both seasons suggests that conducting tissue is an important anatomical component in the response of *O. lagopus* leaves to the dry wet season transition. For fast-growing plantation species, water transport capacity is closely linked to leaf expansion, biomass accumulation, and carbon assimilation potential. The vascular system may therefore be a key anatomical trait connecting external water conditions with plant growth performance.

### 4.2. Carbon-Nutrient Allocation Patterns Revealed by NSC Components and C:N:P Stoichiometric Characteristics

NSC and C:N:P stoichiometric characteristics represent two aspects of plant carbon economy and nutrient allocation. NSC reflects the allocation of photosynthetic products between soluble sugar and starch, whereas C:N:P stoichiometry reflects the proportional relationship between carbon accumulation and N and P nutrition [9,30]. In this study, the seasonal effect on NSC components was weak. Two-year-old *O. lagopus* did not show a significant seasonal fluctuation in the whole carbohydrate pool during the dry wet season transition, but maintained a relatively stable carbon reserve pattern (Figure 2). Roots had higher NSC levels, indicating that belowground organs play an important role in carbon storage and buffering during the early growth stage of *O. lagopus* [31]. For fast-growing tree species, rapid aboveground growth creates a strong carbon demand. Photosynthetic products in leaves may be rapidly used for leaf expansion, stem growth, and export, which can limit large NSC accumulation in leaves [22]. A relatively stable root NSC pool may support basal metabolism in the dry season, fine-root renewal, and growth recovery in the wet season. Although water conditions improve during the wet season, this period also corresponds to the most active growth of *O. lagopus*. Enhanced carbon assimilation is often accompanied by greater growth consumption and assimilate export [22,23]. Therefore, even if leaf soluble sugar or NSC tends to increase, this increase may be offset by rapid growth demand and may not appear as significant seasonal accumulation. Stable NSC concentrations do not indicate inactive carbon metabolism. Instead, they may reflect a dynamic balance between carbon input and carbon consumption. This interpretation is consistent with recent tropical-forest evidence showing that NSC pools and their soluble-sugar and starch fractions vary with plant functional type and site water availability, emphasizing that carbon reserves should be interpreted in relation to life-history strategy and environmental context [32].

Compared with NSC, C:N:P stoichiometric characteristics were more sensitive to seasonal variation, especially P and P-related ratios (Figure 3). This pattern can be understood in relation to soil conditions in the study area. The soil is acidic red soil with abundant Fe/Al oxides, and P is readily immobilized. Plant-available P is therefore strongly constrained by soil chemical properties. Previous studies have shown that increased rainfall can improve soil moisture and P diffusion while also enhancing the leaching of nitrate N, dissolved organic N, and some base cations [33]. Because nutrient leaching was not measured directly in the present study, this process should be regarded as a possible explanation rather than a demonstrated mechanism. For N, leaching losses during the wet season may offset increased supply from mineralization. For P, leaching is relatively weak, but fixation is strong in acidic red soil, and plant-available P depends strongly on water status, rhizosphere pH, and root activity [34]. Thus, wet-season water supply improves nutrient transport and root uptake conditions, but also intensifies leaching and nutrient redistribution. Recent work in tropical coastal forests similarly reported that seasonal rainfall altered leaf soil nutrient concentrations, stoichiometric ratios, and nutrient homeostasis, supporting the view that rainfall-driven changes in nutrient transport and retention can reorganize plant nutrient relationships [35]. In the dry season, leaching is reduced, but soil drying restricts nutrient diffusion and root uptake. The stronger seasonal sensitivity of root P-related stoichiometric parameters in *O. lagopus* may be consistent with water-mediated changes in P availability [36,37]. Low soil moisture in the dry season can reduce P diffusion rates and root uptake capacity, thereby altering root P status and affecting C/P and N/P ratios. Leaf and root N/P ratios in this study were both low. In tropical and subtropical regions with high rainfall and highly weathered soils, N leaching and P fixation may occur simultaneously. Low tissue N/P may be compatible with relative N limitation, but tissue N/P alone does not provide a definitive diagnosis of nutrient limitation. This pattern may be more pronounced during the wet season, when rapid growth increases N demand and strong precipitation may promote N leaching, maintaining low tissue N/P ratios. Likewise, the increase in N/P during the dry season does not necessarily indicate a shift toward P limitation. It is more likely caused by changes in P status or by relative changes in both N and P. Therefore, N/P should be interpreted as an indicator of plant nutritional status rather than a strict diagnosis of nutrient limitation.

Overall, the carbon-nutrient response of *O. lagopus* during the dry wet season transition was asynchronous. NSC components remained relatively stable, indicating a buffering capacity of the carbon pool. In contrast, C:N:P stoichiometric characteristics, especially P-related ratios, changed seasonally, suggesting that nutrient ratios are more sensitive to water conditions and soil processes. This pattern of “stable carbon pool and nutrient ratio reorganization” may be an important physiological feature that supports growth continuity of two-year-old *O. lagopus* under seasonal water fluctuation and nutrient constraints in red soil.

### 4.3. Coordinated Mechanisms Among Leaf Anatomical Structure, NSC Carbon Allocation, and C:N:P Stoichiometry

Correlations among leaf anatomical structure, NSC allocation, and C:N:P stoichiometry showed coordinated structural-carbon nutrient regulation. LT was closely associated with multiple traits related to epidermal, mesophyll, and conducting tissues, indicating that variation in leaf thickness was statistically related to variation in multiple tissue components (Figure 4). PMT and SMT were positively correlated with leaf NSC and N and P concentrations. This pattern suggests that mesophyll development requires both carbon substrate supply and nutrient investment, forming an intrinsic coupling among structural capacity, carbon assimilation, and nutrient demand. XA and MVBA were correlated with leaf soluble sugar, NSC, and SS/ST ratio, indicating that the conducting system is involved not only in water and mineral transport but also in photosynthate export and soluble carbon allocation. This function is important for maintaining the balance between supply and demand in fast-growing *O. lagopus* during the wet season. The first two PCA axes together explained 90.75% of total variance. Samples from the wet- and dry-season sampling campaigns occupied distinct regions of the ordination space, descriptively indicating differences in multivariate trait combinations between the two sampling groups (Figure 5). Wet-season samples were closely associated with UCT, XA, MVBA, soluble sugar, and SS/ST ratio, forming a trait combination characterized by enhanced conducting capacity and carbon allocation. Dry-season samples were associated with SCD, LTL, SMT, leaf and root N/P and C/P ratios, and root starch, forming a trait combination characterized by structural loosening and nutrient ratio reorganization. The correlation between leaf N/P ratio and both SCD and LTL suggests that dry-season water limitation alters internal gas diffusion space and the relative abundance of N and P simultaneously. Given the generally low N/P ratios in leaves and roots, this adjustment more likely reflects nutrient ratio reorganization under relative N deficiency rather than an intensification of P limitation. Significant correlations between leaves and roots in C, N, and P concentrations and their ratios indicate coordinated nutritional status at the whole-plant level. In contrast, weak synchrony of NSC components between leaves and roots confirms functional differentiation in carbon allocation. Leaves mainly support assimilation and export, whereas roots mainly support storage and buffering. Overall, the seasonal adaptation of *O. lagopus* is jointly driven by water supply, nutrient leaching during the wet season, P fixation in red soil, and organ functional differentiation. Wet-season management should reduce the risk of nutrient availability decline caused by N leaching and P fixation. Dry-season management should maintain root-zone water availability to alleviate diffusion limitations. Leaf vascular structure, substomatal chamber traits, root NSC stability, and P-related stoichiometric parameters may serve as potential traits for evaluating seasonal adaptability of juvenile plantations.

## 5. Conclusions

Leaf anatomical traits of juvenile *O. lagopus* showed clear seasonal plasticity during the dry wet season transition. Wet-season leaves were characterized by more developed vascular and midrib structures, suggesting greater conducting demand during rapid growth. Dry-season leaves showed deeper substomatal chambers and higher leaf porosity, indicating changes in internal gas space and mesophyll configuration under water-limited conditions. PCA separated wet- and dry-season samples, with vascular traits and soluble sugar-related variables associated with the wet season, and substomatal chamber depth, leaf porosity and N/P-related traits associated with the dry season. NSC components were mainly characterized by organ differentiation rather than seasonal fluctuation. Roots maintained relatively high and stable carbohydrate levels, suggesting an important role in carbon storage and seasonal buffering. In contrast, C:N:P stoichiometric characteristics were more sensitive to seasonal variation, especially root P concentration and P-related ratios. Leaf and root N/P ratios were consistently below 14, indicating a nutritional status associated with relative N deficiency. Overall, leaf anatomical structure, NSC allocation and C:N:P stoichiometry were closely coordinated. Seasonal adaptation of juvenile *O. lagopus* plantations appears to be regulated by the combined effects of water availability and nutrient leaching during the wet season; possible contributions of nutrient leaching and P fixation remain hypotheses that require direct measurements of soil nutrient availability and fluxes. Vascular traits, substomatal chamber depth, root NSC stability and P-related stoichiometric parameters may serve as potential indicators for evaluating seasonal adaptability in juvenile *O. lagopus* plantations.

## Figures and Tables

**Table 1:** Comparison of leaf anatomical structure between the wet season and the dry season.

Indicators	Dry Season	Wet Season
LT (μm)	173.815 ± 7.097	178.962 ± 9.350
UCT (μm)	1.533 ± 0.108	1.166 ± 0.052 **
LCT (μm)	1.066 ± 0.046	0.965 ± 0.088
UET (μm)	19.567 ± 1.635	22.478 ± 1.221
LET (μm)	9.565 ± 0.439	8.892 ± 0.566
SCD (μm)	51.155 ± 2.822	78.051 ± 5.092 **
PMT (μm)	64.626 ± 1.231	63.553 ± 3.119
SMT (μm)	75.466 ± 4.383	85.406 ± 5.515
P/S	0.875 ± 0.040	0.759 ± 0.045
LTC	0.375 ± 0.011	0.357 ± 0.014
LTL	0.432 ± 0.009	0.477 ± 0.014 *
XA (μm^2^)	406,924.778 ± 78,095.601	43,783.258 ± 7043.908 **
MVBA (μm^2^)	2,764,518.444 ± 184,111.450	1,757,724.971 ± 254,639.313 **

Note: *,** indicate *p* ≤ 0.05, *p* ≤ 0.01, respectively.

**Table 2:** Coefficients of variation and plasticity indices of leaf anatomical traits of *O. lagopus* in the wet and dry seasons.

Indicators	Wet Season		Dry Season	
	Coefficient of Variation	Plasticity Index	Coefficient of Variation	Plasticity Index
LT	0.12	0.32	0.16	0.39
UCT	0.21	0.45	0.13	0.39
LCT	0.13	0.29	0.27	0.59
UET	0.25	0.57	0.16	0.38
LET	0.14	0.32	0.19	0.46
SCD	0.17	0.43	0.20	0.47
PMT	0.06	0.16	0.15	0.35
SMT	0.17	0.44	0.19	0.44
P/S	0.14	0.37	0.18	0.38
LTC	0.09	0.25	0.11	0.26
LTL	0.06	0.19	0.09	0.26
XA	0.58	0.82	0.48	0.86
MVBA	0.20	0.46	0.43	0.72

**Table 3:** Two-way ANOVA of NSC components and C:N:P stoichiometric characteristics across organs.

Factor	Soluble Sugar	Starch	NSC	Soluble Sugars/Starch	C	N	P	C/N	C/P	N/P
Seasons	2.60	1.02	1.38	3.61	4.87 *	1.68	6.22 *	12.99 ***	11.34 **	97.40 ***
Organs	38.20 ***	1.91	33.12 ***	28.45 ***	105.39 ***	329.148 ***	92.54 ***	511.08 ***	67.76 ***	1778.40 ***
Seasons × Organs	1.59	0.56	1.65	1.09	0.31	0.11	1.14	2.15	14.40 ***	1.31

*, **, *** indicate *p* ≤ 0.05, *p* ≤ 0.01, and *p* ≤ 0.001, respectively.

## Data Availability

The raw data supporting the conclusions of this article will be made available by the authors upon request.
